# Intragranular Dispersion of Carbon Nanotubes Comprehensively Improves Aluminum Alloys

**DOI:** 10.1002/advs.201800115

**Published:** 2018-04-19

**Authors:** Kang Pyo So, Akihiro Kushima, Jong Gil Park, Xiaohui Liu, Dong Hoon Keum, Hye Yun Jeong, Fei Yao, Soo Hyun Joo, Hyoung Seop Kim, Hwanuk Kim, Ju Li, Young Hee Lee

**Affiliations:** ^1^ Department of Nuclear Science and Engineering and Department of Materials Science and Engineering Massachusetts Institute of Technology Cambridge MA 02139 USA; ^2^ Advanced Materials Processing and Analysis Center University of Central Florida Orlando FL 32816 USA; ^3^ IBS Center for Integrated Nanostructure Physics Institute for Basic Science (IBS) Sungkyunkwan University Suwon 440‐746 Republic of Korea; ^4^ School of Materials Science and Engineering Shanghai Jiao Tong University Shanghai 200240 China; ^5^ Department of Materials Science and Engineering Pohang University of Science and Technology Pohang 790‐784 Republic of Korea; ^6^ Division of Electron Microscopic Research Korea Basic Science Institute 113 Gwahangno Yuseong‐Gu Daejeon 305‐333 Republic of Korea

**Keywords:** aluminum, carbon nanotubes, creep, in situ transmission electron microscopy, intragranular

## Abstract

The room‐temperature tensile strength, toughness, and high‐temperature creep strength of 2000, 6000, and 7000 series aluminum alloys can be improved significantly by dispersing up to 1 wt% carbon nanotubes (CNTs) into the alloys without sacrificing tensile ductility, electrical conductivity, or thermal conductivity. CNTs act like forest dislocations, except mobile dislocations cannot annihilate with them. Dislocations cannot climb over 1D CNTs unlike 0D dispersoids/precipitates. Also, unlike 2D grain boundaries, even if some debonding happens along 1D CNT/alloy interface, it will be less damaging because fracture intrinsically favors 2D percolating flaws. Good intragranular dispersion of these 1D strengtheners is critical for comprehensive enhancement of composite properties, which entails change of wetting properties and encapsulation of CNTs inside Al grains via surface diffusion‐driven cold welding. In situ transmission electron microscopy demonstrates liquid‐like envelopment of CNTs into Al nanoparticles by cold welding.

## 1 Introduction

Aluminum alloys command a $100+ billion/year world market. The chief advantages of aluminum alloys compared with steels are the higher specific strength, electrical and thermal conductivities, and corrosion resistance. The disadvantages are the cost and high‐temperature capabilities because aluminum has about half the melting point of iron (933.5 K vs 1811 K) and becomes very soft at 500 K or so. This is a mature market, so property changes at a few to tens of percent could change alloy choices within the family, if (a) the increase in cost is not very dramatic and (b) not just one single property, but a comprehensive list of properties are improved. In light of (a) and (b), we will examine the practice of dispersing carbon nanotubes (CNTs) into aluminum alloys. In the previous paper,^1^ we have dispersed CNTs into pure aluminum at ton‐scale and summarized the effects on room‐temperature (RT) properties by a “Taylor‐dispersion hardening” model. The gist is that well‐dispersed CNTs act like forest dislocations. It can harden the metal pretty much like stored dislocation line density in the traditional Taylor work‐hardening model, giving rise to a nonlocal latent hardening of the metal matrix as well as still acting like a composite filler and transmitting load directly as in the traditional composite shear‐lag model. Because mass‐produced multiwalled carbon nanotubes (MWCNT) cost ≈$10^2^ kg^−1^ nowadays, dispersing ≈1 wt% MWCNT into Al matrix would only double the alloy cost which is considered to be a reasonable economic boundary today. Depending on the dispersion and the MWCNT diameter, 1 wt% (equivalent to about 2 vol%) MWCNT would give rise to a dispersed line length density ρ_CNT_ = 10^13^–10^15^ m^−2^. In the present paper, we first show that intragranular MWCNT dispersion is possible in various aluminum alloys (2000, 6000, and 7000 series), and significant improvement in room‐temperature tensile strength can be achieved on top of the well‐known precipitate strengthening mechanism in Al alloys without adverse effect on tensile toughness (**Table** **1**). We then prove that the electrical and thermal conductivities are also slightly improved, which is the first in this field. Then, we show that because mobile dislocations can hardly climb over 1D nanodispersoids (in contrast, they can more easily climb over 0D precipitates, by vacancy flux aided nonconservative dislocation motion), the creep strength of Al alloys is significantly enhanced with its high‐temperature capability raised by 50–100 K, which could be significant for some applications. Lastly, we discuss the underlying reason for the comprehensive enhancements, the key being the good intragranular dispersion we have obtained, that is, the CNTs do not sit only at the grain boundaries (GBs) even though initially that was where they were at, but are also well distributed inside the grains, matching the “Taylor‐dispersion hardening” picture as the bulk dislocation line density is also distributed inside grains. This was in turn rationalized by “cold welding” and rapid surface diffusion of Al atoms in vacuum‐environment mechanical ball milling, which buried the MWCNTs inside the master alloy particles. This cold welding and burying process was revealed by in situ transmission electron microscopy (TEM) experiments under similar vacuum conditions. Our way of producing master alloy and dispersing them are thus key for the comprehensive superiority of the properties over other methods of making metal + CNT nanocomposites.

**Table 1:** Mechanical properties of Al alloy‐CNTs composites

Matrix	CNTs [wt%]	Tensile strength [MPa] (relative change (%))	Yield strength [MPa] (relative change (%))	Young's modulus [GPa] (relative change (%))	Fracture strain [%] (relative change (%))
Pure Al	1	201(±0.58)(51%)	100(±16)(30%)	80(±0.18)(16.5%)	21(±2.2)(−11%)
6000 series	1	227(±3.6)(43%)	143(±4.2)(32%)	–	9.6(±1.8)(−10%)
7000 series	1	264(±1.5)(48%)	–	–	15(±0.6)(−6%)
2000 series	1	295(±13.5)(45%)	192(±7.5)(59%)	–	5.9(±1.4)(−15%)
AlCu/AlSiMg	0.5	429(±6.4)(13%)	383(±7)(16%)	141(±1.6)(0%)	4.75(±0.5)(−36%)
AlCu/AlSiMg (HPT)a)	1	612(20%)	517(8.2%)	70(−4%)	3.14(8.3%)
Dual phase steelb)	0	500	300	−	30–34

^a)^ High‐pressure torsion (HPT);

^b)^ This steel is usually used for automobile body (http://www.worldautosteel.org).

## 2 Materials and Methods

**Figure 1:**
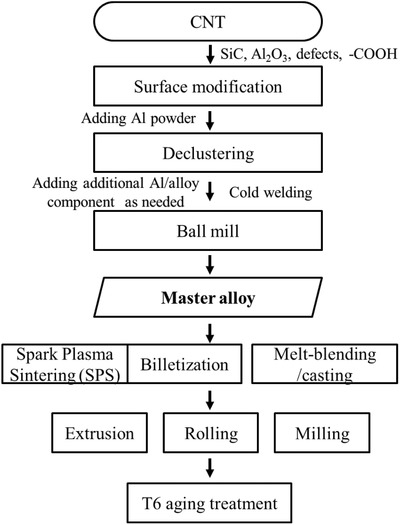
Flow chart of the Al/CNT fabrication process.

**Figure 2:**
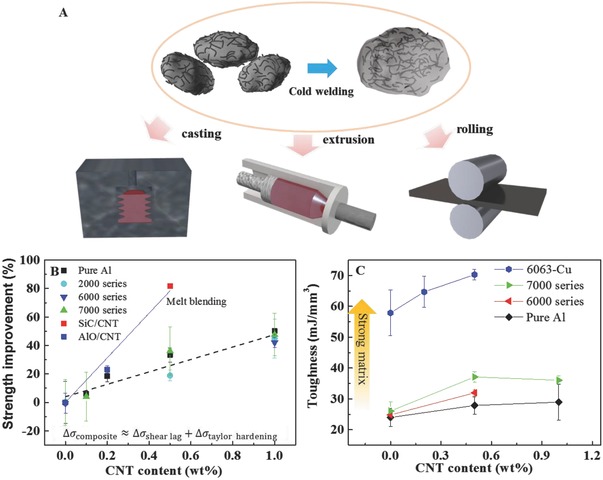
Schematic diagram of the fabrication process of Al + CNT and the mechanical properties. A) Schematic diagram of the declustering of CNT and cold welding. B) Strength improvement from the CNT in a different type of Al alloy and different fabrication methods. The SiC coated CNT was introduced for the melt casting method (red square). Other alloy samples were made using extrusion. C) toughness enhancement of Al alloys at various CNT concentrations.

Good dispersion of CNTs was achieved by the processing flow chart shown in **Figure** **1**. A key intermediate product is the master alloy, a solid powder consisting of 0.1–5 wt% CNTs. Depending on the postmaster consolidation process (which all involves temperature higher than RT) such as melt blending or sintering, premaster surface modification may be required to enhance the wetting and the interfacial strength, that entails coating SiC or Al_2_O_3_ on CNT by thermal decomposition^2^ and microwave treatment (Movie S1, Supporting Information). After the surface modification, we unravel the tangled MWCNTs by a high‐speed blade mixer, which split the clusters into single strands of CNT on the surface of Al particles. The declustered CNTs were then buried inside the Al particles using a planetary ball mill under vacuum. As a consequence of the cold welding to be detailed later, CNTs are encapsulated inside Al grains, creating the master alloy (**Figure** **2**A). The master alloy can then be further processed by spark plasma sintering (SPS), billetization or melting process/casting to create bulk specimen. SPS consolidated the granules and formed the interfacial Al—C covalent bonds. For the melt process, we decorated thin SiC layer on the surface of the CNT to improve the wetting to molten Al.^2^ 5 wt% SiC/CNTs of the master alloy subsequently dissolved into molten Al alloy to become 0.5 wt% CNT (Movie S2, Supporting Information). The consolidated Al + CNT was further shaped by milling, extrusion, and rolling (detailed experimental parameters are described in Table S1, Supporting Information). Previously, we have studied pure Al + CNT.^1^ To see the influence of CNT on alloys in this work, we introduced alloying elements during RT ball milling (mechanical alloying, MA) in Ar. After shaping (extrusion, rolling, and milling), all the alloy composites were subjected to T6 tempering including solution heat treatment and aging treatment, before the mechanical properties were measured.

From our measurements, regardless of the matrix composition and original mechanical properties, the dispersion of CNT in various Al alloys can improve the strength (after extrusion) by 40–50% at 1 wt% CNT (black dash in Figure 2B). Therefore, adding a small amount of CNTs strengthens the materials on the top of existing precipitation hardening. The absolute mechanical properties of different Al alloys are shown in Table 1. The strength increases more after Al_2_O_3_ or SiC surface modification on CNT. ≈80% strength improvement is achieved at 0.5 wt% after melt blending of SiC/CNT (red dot in Figure 1B). SiC has better wettability to Al than carbon, hence it significantly improves the wetting of CNT to molten Al, improving the interfacial bonding and nanoscale dispersion.

## 3 Results

With regard to ductility, although the formability range is slightly reduced, the nonuniform elongation (postnecking) increased, resulting in nearly unchanged tensile strain to failure ε_f_. The toughness, $\int_{0}^{\varepsilon_{f}}\sigma \;d\varepsilon$, increases significantly as the CNT fraction approaches 1 wt% (Figure 2C). The dispersion of CNT significantly increases the absolute toughness of various Al alloys, even in complex multicomponent alloys such as Al–Cu precipitation on the top of A–Si–Mg alloy system, which enables one to measure the highest tensile toughness (Figure 2C, blue line 6063‐Cu). Beyond 1 wt%, the toughness of some composites starts to drop even though the strength continues to increase. This should be because the critical lengthscale for fracture is reached due to more and more severe CNT agglomeration with increasing CNT fraction.^3^

**Figure 3:**
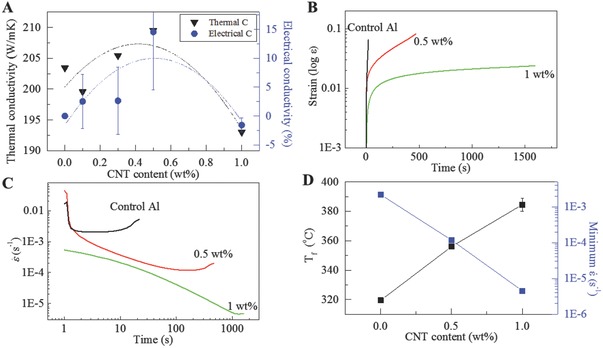
Electrical and thermal properties. A) Thermal conductivity and enhancement of electrical conductivities by adding CNT. B) Strain versus time and C) dε/d*t* at different CNT contents under 300 °C, 70 MPa. D) Fractured temperature (*T*_f_) and minimum strain rate of Al + CNT composite.

Electrical conductivity increases with up to 0.5 wt% CNTs, then gradually decreases (**Figure** **3**A). To the best of our knowledge, no report has shown electrical conductivity improvement by adding carbon nanotubes in metals.^4^, ^5^ Although CNTs with superior electron mobility provides excellent electron transport channels, incoherent interface, and the Schottky barrier between CNT and Al (reported to be ≈0.2 eV^6^) are obstacles. However, good dispersion of CNT inside Al grain enhances electrical transport intra/intergrains. The intimate contact between CNT and Al contributes to lowering the electron transport barrier. As a consequence, CNTs addition into Al slightly increases the electrical conductivity even though they increase mechanical strength. Likewise, Al alloys from the melt processing also show decreased sheet resistance with CNT additions (Figure S7, Supporting Information).^7^ The thermal conductivity has a similar trend with the electrical conductivity. A few reports have shown that the thermal conductivity increases with up to 0.5 wt% of CNTs,^8^, ^9^ similar to our results.

It is conceptually intriguing to compare metal + CNT with other strategies of nanostrengthening, such as oxide‐dispersion strengthened steels, and grain‐boundary strengthened nanocrystalline metals. The unique feature of CNT is that it is 1D, which differs from 0D and 2D strengtheners. At high temperatures, a small amount of diffusion would allow dislocations to climb over 0D dispersoids/precipitates. But they would find it nearly impossible to climb over 1D CNTs that cross its path which have an aspect ratio as long as 10^3^. And unlike 1D forest dislocations which can be annihilated by dislocation reactions (recovery) and recrystallization, the 1D CNTs cannot be annihilated this way. They also do not coarsen like the grains do, since CNTs are not required to form a percolating network like the GBs, and there is also no significant thermodynamic driving force for CNT dispersion to coarsen like the grains when there is good wetting with metal. Thus, we expect the metal + CNT to have superior high‐temperature creep strength than corresponding 0D strengthened alloys. Also, fracture favors percolating, 2D flaws because elastic fracture mechanics prefers planar cracks (oblate over prolate), so grain‐boundary strengthening, although highly effective at RT as shown by the Hall–Petch relation, also tends to embrittle the material macroscopically due to the extreme‐value statistics nature of fracture. Furthermore, at high‐temperature, grain boundary diffusion can greatly accelerate creep.

To evaluate the high‐temperature creep strength, we measured the creep strain rate of Al + CNT at 300 °C (Figure 3B) or 573.15 K. As is well known, aluminum melts at *T*_M_ = 933.5 K and usually becomes very soft above *T*_M_/2. The strain rate was measured at 300 °C using dynamic mechanical analyzer (DMA) under 70 MPa engineering stress (dead load) for all samples. DMA requires very small sample (only few milligram) quantity and size and has good sensitivity in applying accurate force (10 µN) and temperature (±0.1 °C). It detects a very small range of displacements (1 nm). Thus, it is very efficient in evaluating creep properties. The measurement time is only a few minutes to hours. The pulling direction is aligned with the CNTs in the thin film geometry, which afford more effective pinning of dislocations climbing in transverse directions. The creep strain rate $\dot{\varepsilon }$ decreases by three orders of magnitude by adding 1 wt% (Figure 3C). The strain rate of pure Al + 1 wt% CNT in stage II is ≈10^−6^ s^−1^ at 300 °C and 70 MPa, similar to the reported ≈20 vol% SiC whisker/Al6063.^10^

Also, the creep fracturing of the specimen at high temperatures was measured using standard testing equipment (Applied Test Systems, Inc.) calibrated by American Society for Testing and Materials (ASTM) E4 with a dead load that corresponds to 50% of the RT yield strength of this material. The temperature was measured by welding two k‐type thermocouples on the both ends of grip holder for accurate temperature estimation. The 300 mm length of the heater is big enough to cover the entire sample (50 mm) including grip holders. We use the linear variable differential transformer as an extensometer to measure the displacement when the temperature increases. The control pure Al creeps as the temperature rises at 100 °C h^−1^ and is fractured at 320 °C (Figure S6, Supporting Information). In contrast, the 0.5 and 1 wt% CNT + Al showed reduced creep rate and fractured at significantly higher temperatures. The creep fracture temperature (*T*_f_) increases linearly with CNT addition (Figure 3D). The high‐temperature ability is almost enhanced by 70 °C with 1 wt% CNT dispersion, which is very significant (0.08*T*_M_) in both relative and absolute terms. It means the range of applications of aluminum alloys may be significantly expanded.

## 4 Discussion

Metal + CNT composites have been extensively studied during the last two decades, but most efforts were focused on RT strength.^11^, ^12^ The key to establishing the comprehensive high performance of alloy + CNT composites is the “good dispersion” of CNTs, which entails several aspects. It is well appreciated that brittleness can be reduced by disaggregating inclusions and keeping the characteristic length scale of an individual inclusion at below tens of nanometers.^3^ One certainly does not want voids between CNTs and metal matrix, and hopes the dispersion of CNTs does not significantly delay the sample from achieving 99+% of the theoretical density. Lastly, it has been shown empirically that nanoprecipitation/particles in metals and alloys should be better situated in the grain interior (intragranular) than on the GB, in order to increase mechanical strength while preserving/extending the ductility.^13^, ^14^ This is probably because GBs are 2D and also naturally form a percolating network, while CNTs are 1D and precipitate/particles are 0D. Fracture favors percolating 2D flaws (cracks are 2D) by virtue of elasticity, whereas interfacial debonding along 1D and 0D flaws, even if it happens, are less dangerous from a stress intensity factor point of view. GBs themselves are already excellent flow strengthening agents by forcing dislocation slip to change direction.^15^ Adding CNTs right on GBs will likely further strengthen the flow strength, but may also cause the GB cohesion strength to go down, and once some debonding has happened fracture may propagate much quickly along a 2D percolating GB network. So, intragranular dispersion of CNTs should be recommended relative to intergranular dispersion of CNTs.

However, for the metal + CNT nanocomposite, besides conventional problems mentioned above, the oxide layer on metal, the difference in surface tension between metal and CNTs and wetting of CNTs, and bonding strength of metal + CNT interface are other critical factors to be overcome. For example, the oxide layer formed on the surface of metal is detrimental to uniform dispersion of CNTs in metal matrix since mechanically robust nanosize oxide layer acts as a strong barrier to the CNT trying to penetrate inside the grain.^16^, ^17^, ^18^ Such an oxide layer segregates CNTs from being uniformly distributed inside the metal matrix, consequently limiting its dispersion. Even if the oxide layer can be broken, the high surface tension of metals and poor adhesion of carbon would resist mixing CNTs in metal.

Intragranular dispersion in metals and alloys were often induced through thermal precipitation^14^, ^19^ and liquid phase processing.^20^ However, these methods are limited due to the thermochemical stability requirement on the particles. In comparison to these methods, mechanical alloying (MA) provides more freedom to choose the matrix and particles.^21^ Deformation and cold welding of metals at nanoscale are much different from those of the bulk. Surface allows fast atomic diffusion.^22^ Especially, when the metal particle size reaches near 10 nm, crystalline metals show viscous deformation and thus the envelopment easily occurs without applying much external force, showing liquid‐like behavior even though the interior remains crystalline.^23^ This liquid nature of metallic surface provides an opportunity to mix thermodynamically unstable/incompatible phases such as CNT.

**Figure 4:**
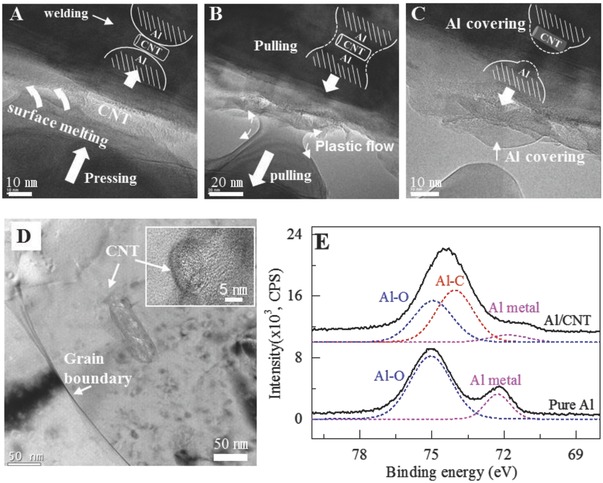
In situ TEM observation of the mechanism of CNT embedding in Al under no‐oxidation conditions. A–C) CNT embedment via surface melting process: A) surface melting‐driven cold welding on the contact area, B) Al disconnected through plastic flow, and C) residual Al covered on the top of CNT. See Movie S3 in the Supporting Information. D) TEM observation for the nanoscopic dispersion shows CNTs in the Al grain interior (inset: The intact wall structure of intragranular CNT, 3.3 Å interlayer distance of the graphitic layer). E) Al 2p peak in XPS of pure Al (bottom) and Al + CNT 10 wt% at 600 °C during SPS (top).

In situ TEM observations verify the liquid‐like envelopment mechanism of the intragranular dispersion of CNT in Al via cold welding derived from rapid surface diffusion of Al. The oxide‐free Al was prepared under in situ TEM by applying tension to sample until fracture occurred (Figure S8, Supporting Information). We then transferred a CNT on the bare Al surface by manipulating the sample with a piezo‐actuator equipped in Nanofactory scanning tunneling microscopy (STM)‐TEM holder. The two bare surfaces of Al started to weld via surface diffusion after contacting each other, to minimize the surface energy.^23^ The CNT was covered by Al (**Figure** **4**A). Since the cold welding forced the two Al pieces to squeeze the CNT into the middle and became one piece, necking and fracture took place as they have pulled apart (Figure 4B). The fracture point was different from the welded interface, leaving a fraction of Al still attached to the lower part of the upper Al, covering the CNT (Figure 4C and Movie S3, Supporting Information). By repeating this process, the CNT was totally embedded into the Al matrix. This shows good wetting of Al onto CNT when there is no oxide layer.

We quantitatively analyze the surface diffusivity from in situ TEM by taking the flattening time of a curved surface after the coverage of CNT via cold welding (Figure S12, Supporting Information).^24^, ^25^, ^26^ The surface diffusivity *D*_s_ can be estimated by the following equation^26^

(1) $$D_{s}=-\frac{Sk_{B}T}{\nu \gamma_{M}\Omega^{2}}{\left(\frac{\lambda }{2\pi }\right)}^{4}$$

where *S* is the slope from the natural logarithm of height versus a linear function of time in Figure S13A in the Supporting Information, Ω is atomic volume (0.0166 nm^3^ per atom), *T* is room temperature (298 K), ν is the surface atomic density (ν_(111)_ = 0.1 atom nm^−2^), and λ is the segment length in the rest position (24 nm in this case). The surface diffusivity (*D*_s_) is estimated to be 3.27 × 10^−11^ cm^2^ s^−1^ at room temperature. The value is very close to the empirical diffusivity of the surface, 1.8 × 10^−11^ cm^2^ s^−1^ at room temperature.^24^ The activation energy of the atomic self‐diffusion was previously reported in Table S2 in the Supporting Information.^24^ The surface diffusivity *D*_s_ is higher than those of GB, dislocation, and bulk.^24^ Since the surface state is dynamic instead of the rigid in nanoscale, atoms easily diffuse through surface, like a viscous “2D liquid.”^23^ The fluid nature observed in in situ TEM agrees reasonably with our MA ball milling experiment. Ball milling is a dynamic condition. Impact and shearing between ball–ball or ball–container continuously deform and refine the Al nanoparticles, exposing bare surfaces. The volume fraction of surface atoms increases as the particle size decreases. At below 100 nm, 1 vol% of the atom occupies the surface which is comparable to the volume fraction of the CNTs (Figure S13C, Supporting Information).

To induce this diffusion‐driven cold welding in MA, free random movement of atoms on the surface is an essential condition. Thus, the formation of the oxide layer in Al particles needs to be prevented. Chen et al. reported that purging of argon into the ball mill container would not inhibit oxidation of Al during ball milling.^27^ To satisfy the required condition to enhance the atomic diffusion above, we designed ball milling inside the Ar‐filled glove box (<0.1 ppm O_2_, <0.1 ppm moisture). Al was reported to be oxidized by exposing to 10^−6^ torr vacuum for 100 s.^28^ 0.1 ppm of oxygen is two orders higher concentration of oxygen than 10^−6^ torr, hence the glove box environment is equal to 1 s exposure in the same vacuum condition. As the ball milling is a dynamic condition where the frequency of collision event of the ball is >10^4^ s^−1^,^29^ 1 s is more than enough to allow for anchoring CNT on bare Al surface to induce surface diffusion and cold welding before reoxidation (≈10^11^ CNTs are embedded each second). Furthermore, the temperature inside of the ball mill container is ≈200 °C, which makes the diffusivity three order higher than that at RT.^30^ This condition makes CNTs completely feel the liquid‐like aluminum on the surface, with plenty of opportunities to get enveloped. For better cold welding, we disaggregated the CNT clusters and located them on the surface of Al particles using a high‐speed blade mixer (decluster). The declustered individual CNTs on Al surface were further ball milled to induce cold welding and encapsulation of CNTs into Al. After declustering of CNTs, the number of CNTs on the Al is ≈90 µm^−2^ which is small enough to enable the embedding of all the CNTs into the Al matrix during the ball milling. This step creates a high concentration of CNTs in the master alloy which is essential for the tons‐scale industrial application. The encapsulated CNTs in the master alloy have an intimated interface through Al—C bonding^31^ and Si—C bonding. It consequently allows dispersion into macroscopically molten Al alloys (see Supporting Information Movie S2) without significant segregation of CNTs.^1^, ^2^

To verify the nanodispersion of CNTs (individual CNTs) in bulk Al + CNT composites, confocal Raman and TEM were used. The CNTs were mostly located inside the grains, as shown in the TEM image (Figure 4D). This result contrasts starkly with ball milling without controlling the environmental oxidation, where CNTs are strongly localized at the Al particle boundaries due to the limited atomic diffusion near the Al surface oxide layer (Figures S9 and S10, Supporting Information). Consequently, less improvement in the tensile strength and more degradation in fracture strain were observed (Figure S11, Supporting Information). This shows the comprehensive enhancement of properties is intimately related to the surface condition of Al during ball milling in creating the master alloy.

The linear increase in Young's modulus at CNT concentrations up to 1 wt% (Figure S16, Supporting Information) clearly indicates efficient load transfer through the formation of a strong interfacial bonding between Al and CNT surfaces. The Al—C covalent bonds were confirmed from X‐ray photoelectron spectroscopy (XPS) spectra (Figure 4E). A large portion of the Al—C peak was generated in the Al + CNT composite, whereas aluminum oxide peak was not as prominent as that of pure Al. The related Al_4_C_3_ peaks in the X‐ray diffraction (XRD) patterns and the blueshifting of G‐band in Raman are also clear evidence of strong interfacial bonding (see the characterization of the interfacial bonding in the Supporting Information).

## 5 Conclusion

**Figure 5:**
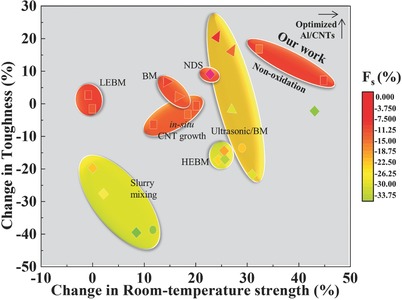
A plot of change in room‐temperature tensile strength, toughness, and fracture strain (F_s_) by adding 1 vol% of CNTs.

The quantitative contribution of CNTs to mechanical strength enhancement can be analyzed using the strengthening efficiency (*R*), $R\equiv (\sigma_{c}-\;\;\sigma_{m})/\upsilon_{c}\sigma_{m}$, where σ_c_ and σ_m_ are the tensile strengths of the composite and matrix, respectively, and υ_c_ is the volume fraction of CNTs to metal, is the change in strength by adding unit volume of CNTs.^32^ The strengthening efficiency is generally influenced by the degree of CNT dispersion and interfacial strength. **Figure** **5** summarizes the relationship of these properties with different materials manufacturing methods. Since an intragranular nanodispersion strategy of CNTs in Al matrix leads to enhanced mechanical strength with a tenable ductility, both strengthening efficiency and toughness are consistently enhanced with an addition of CNTs. All our data for the different alloys show the best values for specific toughness versus specific strengthening efficiency among reported literature. The significantly improved high‐temperature creep capability (by 70 °C or 0.08*T*_M_), the excellent electrical and thermal conductivities, the acceptable cost, and the wide applicability of this method to both pure Al and Al alloys (2000, 6000, and 7000 series) mean the intragranular dispersion of 1D nanowires and nanotubes is a new paradigm for making high‐performance structural materials.

## 6 Experimental Section

*Intragranular Dispersion of CNT in Al*: The master alloy was made by RT ball milling in Ar environment. Subsequently, it can be further downblended by impeller‐driven mixing in the molten state in the same alloy matrix (see Movie S2, Supporting Information). After the surface modification, the tangled MWCNTs were unraveled by a high‐speed blade mixer (VM0104, Vita‐Mix, USA) for 20 min at max. 37 000 rpm, which split the clusters into single strands of CNT on the surface of Al particles. The declustered CNTs were then buried inside the Al particles using a planetary ball mill for 30 min at 250 rpm in a glove box under less than 0.1 ppm of oxygen and moisture to prevent oxidation. As a consequence of the cold welding, CNTs are encapsulated inside Al grains, creating the master alloy (Figure 2A). The master alloy can then be further processed by SPS, billetization, or melting process/casting to create bulk specimen. SPS consolidated the granules and formed the interfacial Al—C covalent bonds. the sintering conditions were optimized to yield a density greater than 99% of the theoretical value by controlling the temperature and time. The high relative density was obtained due to the encapsulation of CNTs inside the Al particles, i.e., no void volume was produced by CNT agglomeration on the particle boundaries. Microstructural observations demonstrate that the oxide layer on the Al granule surfaces was successfully disintegrated by the SPS process, forming discrete oxide nanoparticles. For the melt process, thin SiC layer was decorated on the surface of the CNT to improve the wetting to molten Al.^2^ 5 wt% SiC/CNTs of the master alloy subsequently dissolved into molten Al alloy to become 0.5 wt% CNT while stirring with a graphite impeller in a vacuum (10^−3^ torr). The consolidated Al + CNT was further shaped by milling, extrusion, and rolling (detailed experimental parameters are described in Table S1, Supporting Information).

*Fabrication of CNTs + Al Alloy Composite*: Cu (1 wt%) was added for the 2000 series and Zn (5.6 wt%), Mg (2.5 wt%), and Cu (1.6 wt%) were added for 7000 series to pure Al. For the 6000 series alloy‐CNT composite, the Al 6063 ingot was atomized to powder and proceeded with the same procedure. After shaping (extrusion, rolling, and milling), all the alloys were subjected to T6 tempering including solution heat treatment and aging treatment, before the mechanical properties were measured (Table S1, Supporting Information).

*Characterization of Mechanical/Thermal/Electrical Properties*: Mechanical properties were characterized using an ultimate tensile tester (Landmark 25 kN, MTS, USA) and a micro‐Vickers hardness tester (HM‐211, Mitutoyo, Japan). The tensile specimen was prepared using a mechanical mill with a 6 mm gage diameter and a gage length of 25 mm (E8/E 8M‐08, ASTM). The tensile test was performed at a speed of 2 mm min^−1^. The hardness test was conducted on a cross‐section of the specimen using a load of 100 g for 10 s. The thermal conductivity was determined by laser flash analysis. The electrical conductivity was determined by measuring the electrical resistance of a metallic wire as well as four‐probe method to measure the sheet resistance. The high‐temperature creep properties were characterized by applying a dead load and measuring the strain rate in the DMA (Q800, TA instrument), and creep fracture temperature (*T*_f_) measurement using conventional dog bone shape specimen with pin–hole on the grip part after 400 °C for 32 h annealing treatment. For the DMA measurement, the Al + CNT composite was thinned down to 100 µm by cold rolling and applying 70 MPa engineering stress at 300 °C. For the *T*_f_ measurement, a pin‐loaded tensile specimen was made modified from ASTM E8 with 50 mm total length, 20 mm gauge length, and 2 mm of thickness. The temperature was linearly increased until fracture occurred. The applied load in the *T*_f_ experiment was half of RT yield strength of the respective material, where the RT yield strength was converted from RT Vickers hardness.

*In Situ TEM Observations*: An in situ TEM experiment was conducted to verify the nanoscale mechanism of atomically surface‐diffusion driven cold welding of Al for dispersing and locating the CNT inside Al grain. Nanofactory STM‐TEM holder equipped with 3D piezo‐manipulator was used for this experiment (Figure S8A, Supporting Information, left picture). T‐shaped Al sample with thickness ≈100 nm was prepared using focused ion beam (FIB) and transferred to the tip of a W probe and welded by Pt electron beam deposition inside a scanning electron microscopy (Helios Nanolab 600 Dual Beam FIB Milling System) as shown in Figure S8B in the Supporting Information. W probe with a hook‐shaped tip was prepared using the same system to pull the Al sample in situ inside TEM (JEOL 2010F). The oxide‐free Al was prepared inside in situ TEM by applying tension to the FIB‐cut sample until fracture (Figure S8A, right, Supporting Information). A CNT was then transferred on the bare Al surface by manipulating the sample with a piezo‐manipulator as shown in Figure S8C,D in the Supporting Information.

## Conflict of Interest

The authors declare no conflict of interest.

## Supporting information

Supplementary

Supplementary

Supplementary

Supplementary

Supplementary
