# Supplementary material for: Intragranular Dispersion of Carbon Nanotubes Comprehensively Improves Aluminum Alloys
Source: Adv Sci (Weinh). 2018 Apr 19;5(7):1800115. doi: 10.1002/advs.201800115 (PMC6051391; doi:10.1002/advs.201800115)
Supplement: Supplementary file 1 — Supplementary [file ADVS-5-1800115-s001.pdf]

Copyright WILEY-VCH Verlag GmbH & Co. KGaA, 69469 Weinheim, Germany, 2018.

ADVANCED SCIENCE
Open Access

Supporting Information

for *Adv. Sci.,* DOI: 10.1002/advs.201800115

Intragranular Dispersion of Carbon Nanotubes Comprehensively Improves Aluminum Alloys

Kang Pyo So, Akihiro Kushima, Jong Gil Park, Xiaohui Liu, Dong Hoon Keum, Hye Yun Jeong, Fei Yao, Soo Hyun Joo, Hyoung Seop Kim, Hwanuk Kim, Ju Li, and Young Hee Lee**

SUPPLEMENTARY INFORMATION

Materials and Methods

A. *Fabrications*

Sample preparation

The uniform dispersion of CNTs into Al grains was achieved from the three main factors, (i) the CNT declustering, (ii) cold-welding and (iii) interfacial bonding. The flow chart of processes is shown in Fig. 1. Depending on the post-process such as melt blending and sintering, type of the surface modification is required to enhance the wetting and the interfacial strength. The surface of CNT can be modified by the organic functional group, defects or inorganic layer such as SiC and Al_2O_3.

The SiC layer decorated by thermal decomposition in previous report.[1] The diameter and length of the multi-walled (MW) CNTs (CM95, Hanwha Nanotech, Korea) were in the range of 10–30 nm and a few tens of micrometers, respectively. A 1:1 atomic ratio mixture (7:3 by weight ratio) of Si powder (325 mesh, 99%, Aldrich) to MWCNTs were mechanically crushed using a planetary ball miller (Pulverisette 6, Fritsch, Germany) for 10 h at 230 rpm with 5 mm zirconia balls. Additional MWCNTs were added to this crushed mixture. Different mixing ratios (3:5, 6:5, 12:5 and 20:5) of the crushed mixture to MWCNTs were investigated. To form SiC on the MWCNT surfaces (SiC/CNT), high-temperature annealing was performed at 1300 °C for an hour in a vacuum induction furnace.

The Al_2O_3 particles are decorated by the microwave treatment after anchoring aluminum precursor. 1~5g of aluminum tri-acetyl acetonate were decorated in 1 g of MWCNT in 0.5g poly acrylic acid in water. After coating of the aluminum precursor on the MWCNT, the samples are completely dried and place into microwave oven under argon atmosphere. Thermal spikes were observed during the microwave irradiation for 5 min. After the microwave treatment, unform coating of Al_2O_3 were seen on the surface of CNT as shown in Fig. S1

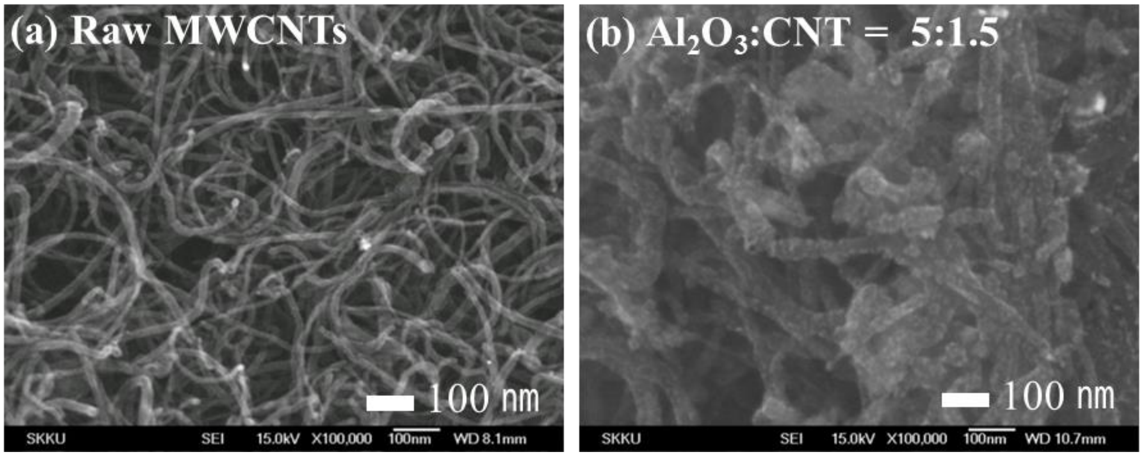

Fig. S1 SEM images after Al_2O_3 decoration on CNTs. (a) Raw MWCNTs and (b) decoration of Al_2O_3 particles on CNTs after microwave treatment.

The declustering process of CNT on the surface of Al particles was performed by a high-speed blade mixer (VM0104, Vita-Mix, USA) to unravel the tangled multiwalled carbon nanotubes (MWCNTs), (CM95, Hanwha Nanotech, Korea) for 20 min at max. 37,000 rpm (Fig. S2B and Fig. S3C). The declustered CNTs were cold-welded using a planetary ball miller (J.E. Powder, Korea) for 30 min at 250 rpm to produce the master alloy, as shown by the Al/CNT granule in Fig. S2D and Fig. S2D. The process was completed in a glove box (M.O. Tech, Korea) under less than 1 ppm of oxygen and moisture to prevent oxidation. For the CNT volume calculation, a CNT density of 1.3 g/cm^3 was used. The concentration of CNTs was varied from 0.1 wt% to 5 wt%. As a consequence of the cold-welding, CNTs are encapsulated in Al particles that were further consolidated under 40 MPa with spark plasma sintering (SPS, 50 t, 50 kW, Eltek, Korea) to form interfacial Al–C covalent bonds at 560 °C for 15 min (Fig. S2E). The bulk Al/CNT composites were extruded from 2.5 mm to 8 mm in diameter with an extrusion ratio of 9:1 at 550 °C (Fig. S2E and Fig. S3E).

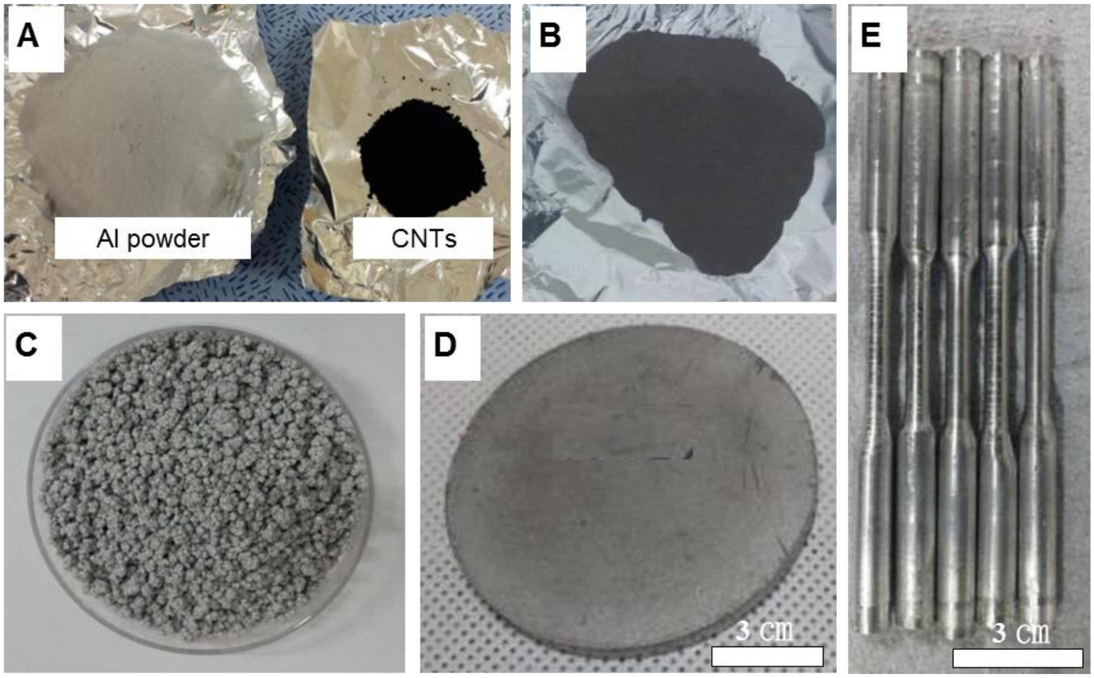

Fig. S2 Schematic illustration for fabricating Al+CNT composite via surface melting-driven cold welding and sample photos for each process. (A) The Al powder and the pristine CNT powder, (B) after the declustering process of 2 vol% CNT, (C) after encapsulation of 0.4 vol% CNT, (D) after SPS of Al/CNT 0.4 vol%, and (E) after extrusion of Al/CNT 0.4 vol%. The change of powder color from grey to black indicates that individual CNT was attached on the surface of Al particles after declustering process as (B). CNTs were encapsulated during mechanical ball mill in a glove box, forming 2 mm diameter granules, as shown in (C). The color was converted into grey, ensuring encapsulation of CNTs inside Al particles. The complete CNTs encapsulation was further verified by bright silver color after extrusion (E).

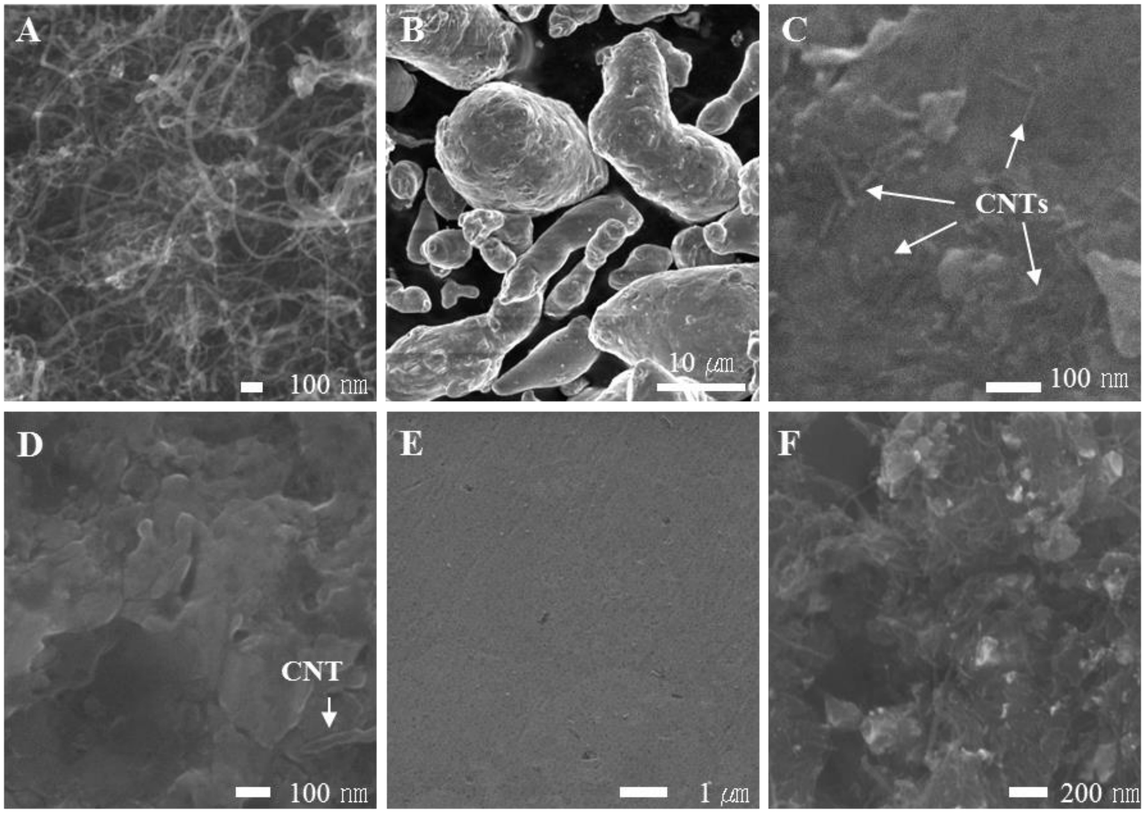

Fig. S3 SEM images for each step. (A) Raw CNTs, (B) raw Al powder, (C) Al/CNT composite after 2 vol% CNT declustering process. Individual CNTs were visible on the surface of Al particles. (D) Master alloy after encapsulation of 0.4 vol% CNTs. (E) Al/CNT composite after SPS and extrusion, and (F) CNTs embedded inside Al, which appeared after acid etching. The diameter and length of MWCNTs were in the range of 10~30 nm and a few tens micrometers, respectively (A). The purchased gas-atomized aluminum powder was filtered by 200 meshes to have particle sizes less than 50 microns, as shown in (B). Individual CNTs were observed on the surface of Al particles after declustering process, as shown in (C). The declustered CNTs were embedded inside Al particles after oxygen-free mechanical pulverization-assisted encapsulation process, as shown in (D). Al particles were fractured. The bare surface was exposed without oxidation during mechanical pulverization. The fractured Al particles were coalesced to each other during further pulverization process, forming CNT-embedded Al granules. The CNT morphology (1 μm length) was well maintained and detected after the final process, as shown (F).

To see the influence of CNT in the precipitation strengthened alloys, we prepared Al alloys/CNT composites. The alloy elements were introduced during ball milling (mechanical alloying). We add Cu (1 wt%) for the 2000 series and Zn (5.6wt%), Mg (2.5 wt%), Cu (1.6wt%) for 7000 series to pure Al. For the 6000 series alloy-CNT composite, we atomized the Al 6063 ingot to powder and proceeded with the same procedure. All the alloys were subjected to T6

tempering including solution heat treated and aging treated. The detailed experiment parameters are described in Table S1.

Table S1. Fabrication process and characterization of Al alloy CNT

Matrix	Interface	Dispersion process	Bulk formation	Shape forming	Heat treatment	Properties
Pure Al	Al-C	mixer/ball mill	SPS	Extrusion	N/A	Tension, hardness, electric/thermal conductivity
				Extrusion/rolling	Annealing 350°C/5h	Creep/T_f
	Si-C[1]	mixer/ball mill	Melt blending	extrusion	N/A	Tension
	Al_2O_3	mixer/ball mill	Melt belding	extrusion	N/A	Tension
2000 serise	Al-C	Multi-step Ball mill	Compacting	extrusion	Induction heating 550 °C/1h, natural aging	Tension
6000 serise	Al-C	mixer/ball mill	SPS	extrusion	530°C/4h, 175°C/8h	Tension
7000 serise	Al-C	mixer/ball mill	SPS	extrusion	480°C/24h, liquid N2 quenching, 100 °C/5h	Tension
AlCu/AlSiMg	Al-C	mixer/ball mill	SPS	extrusion	530°C/4h, 175°C/8h	Tension
AlDC 12.2	Si-C[2]	Ball mill	Melt blending	casting	N/A	Electric resistivity

SPS optimization

We optimized the sintering conditions to yield an Al+CNT composite with a density greater than 99% of the theoretical value by controlling the temperature and time, as shown in Fig. S4A and B, respectively. To achieve a relative density of 99%, a sintering temperature of 560 °C was used with a sintering time of 15 min at a pressure of 46 MPa. The increased relative density was easily obtained because CNTs were encapsulated inside the Al particles, i.e., no void volume was produced by CNT residence in the particle boundaries. Microstructural observations demonstrate that the oxide layer on the Al granule surfaces was successfully disintegrated by the SPS process, forming discrete oxide nanoparticles, as shown in Fig. S5A to C.[3,4]

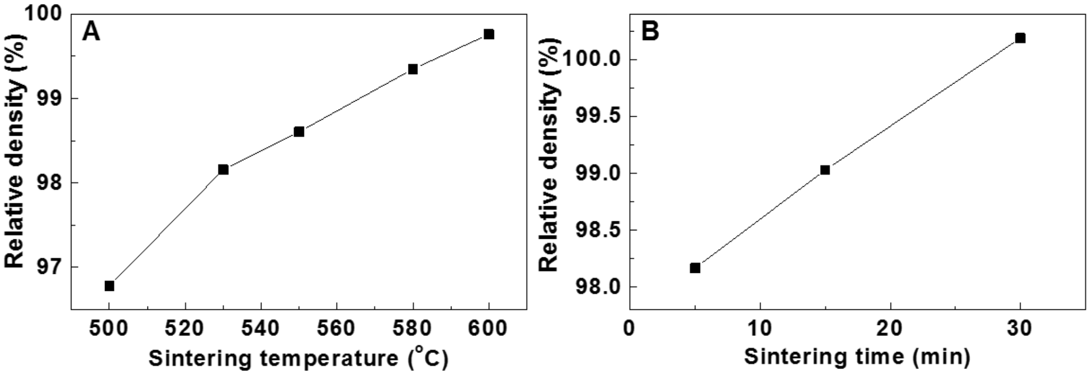

Fig. S4 Sintering process-dependent relative density compared to that of the Al/CNT composite. (A) The sintering temperature- and (B) sintering time-dependent relative densities. The reference density was calculated by, $\rho_{\mathrm{Ref.}} = \frac{\rho_{CNT}\rho_{Al}}{\rho_{CNT}m_{Al} + \rho_{Al}m_{CNT}}$ where ρ_{CNT}, ρ_{Al}, m_{CNT} and m_{Al} are CNT density, Al density, CNT weight fraction and Al weight fraction, respectively. A CNT density of 1.3 g/cm^3 was used. The relative density was obtained from experimental density divided by the reference density. The increased relative density to nearly to 100% was easily obtained because CNTs were encapsulated inside the Al particles, i.e., no dead volume was produced by CNT residence in the particle boundaries. To achieve high-density more than 99 % relative density, 580 °C and 15 min of the sintering temperature and time were used.

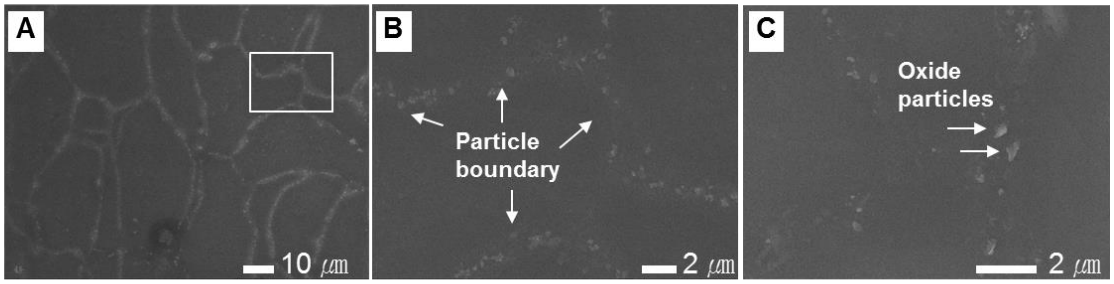

Fig. S5 Microstructure observations after SPS at 580 °C for 15 min with 2 vol% CNT. (A) A particle boundary trace indicated by white lines, (B) enlarged in (A), and (C) oxide nanoparticles. The oxide layer on the surface of Al granules was formed after encapsulation during sample transfer for SPS. Note that CNTs were already well dispersed inside Al granules. Microstructural observations demonstrate that the oxide layer on the encapsulated Al granule surfaces was successfully disintegrated by the SPS process, forming discrete oxide nanoparticles.

B. High-temperature capability test.

The Al+CNT samples are rolled down to 2mm by cold rolling. We made the pin-loaded tensile specimen modified from ASTM E8 with 50mm of total length and 20mm of gauge length. The applied force was determined by half of yield strength where the yield strength was converted from Vickers hardness. The parameters are described in Table S2. We use ASTM E4 standard calibrated equipment (ATS applied test system INC.). All the sample were heat treated under 400°C for 32hrs to release the internal micro strain during the sample preparation. The heating rates were 100°C/hr up to fracture. The elongation vs temperature is shown in Fig. S6. The fractured temperatures (T_f) are determined after the final fracture.

Table S2. Applied force for high-temperature capability

**Samples**	**Hardness (Hv)**				**Av.**	**Yield st. (MPa)**	**0.5 σ_y**
Control Al	58	56	57	56	56(±0.5)	109	54
0.5 wt%	70	66	67	73	69(±1.6)	124	62
1 wt%	78/75	89/85	66/72	79/74	77(±2.6)	134	67

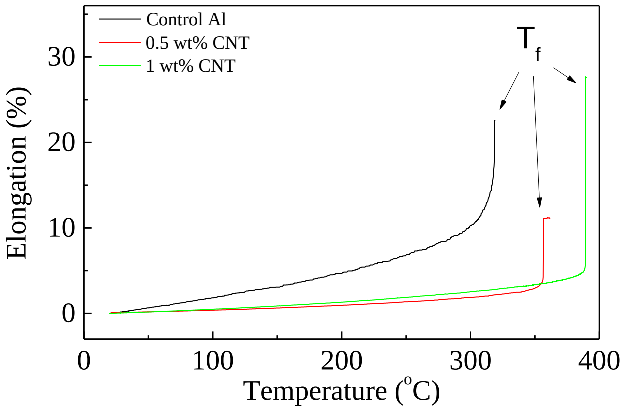

Fig. S6 Elongation of Al+CNT at a different temperature.

The strain rates were measured in the Dynamic mechanical analyzer (DMA, Q800, TA instrument). The sample was thin down to 100um by rolling. Thin films were used to measure the strain rate. All the films were heat treated 350°C for 5hrs.

C. Electrical and thermal conductivity.

We have used the four probe method to measure electrical conductivity after extrusion 2 mm of diameter. To measure accurate electrical conductivity, we extrude the length to 1 m. For the comparison purpose, we fabricate Al alloy +CNT composite by using melt-processing with the ALDC 12.2 die-casting alloy as a matrix. We measure the 4 probes electrical resistance on the surface by using Van der Pauw method after polishing the surface enhance the contact. The measured area is 1 cm^2 on the surface of Al+CNT composite after casting. The resistance is shown in Fig. S7. The thermal conductivity was characterized by Laser flash analysis (LFA) after shaping 1cm x 1cm with 1mm thickness samples.

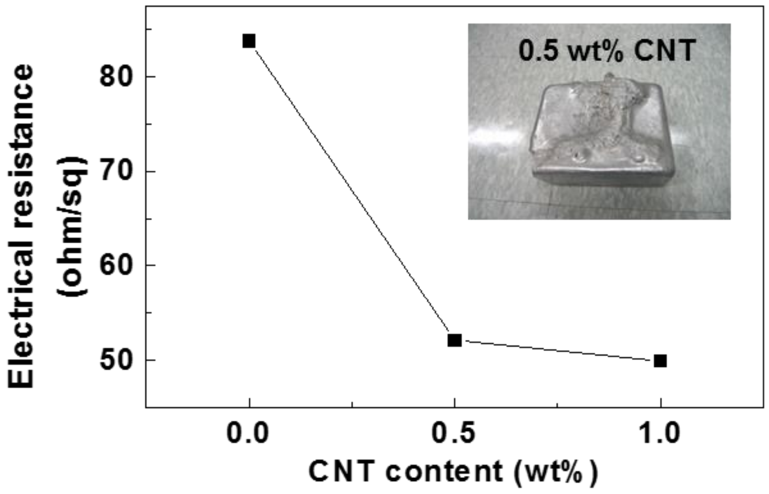

Fig. S7 Surface resistant of the Al+CNT composite.

D. *In-situ* TEM observations

We conducted an *in-situ* TEM experiment to verify the nanoscale mechanism of atomically surface-diffusion driven cold-welding of Al for dispersing and locating the CNT inside Al grain. Nanofactory STM-TEM holder equipped with 3D piezomanipulator was used for this experiment (Fig. S8A, left picture). T-shaped Al sample with thickness ~100 nm was prepared using focused ion beam (FIB) and transferred to the tip of a W probe and welded by Pt electron beam deposition inside a scanning electron microscopy (Helios Nanolab 600 Dual Beam FIB Milling System) as shown in Fig. S8B. W probe with a hook-shaped tip was prepared using the same system to pull the Al sample *in-situ* inside TEM (JEOL 2010F). The oxide-free Al was prepared inside *in-situ* TEM by applying tension to the FIB-cut sample until fracture (Fig. S8A, right). We then transferred a CNT on the bare Al surface by manipulating the sample with a piezo-manipulator as shown in Fig. S8C and D.

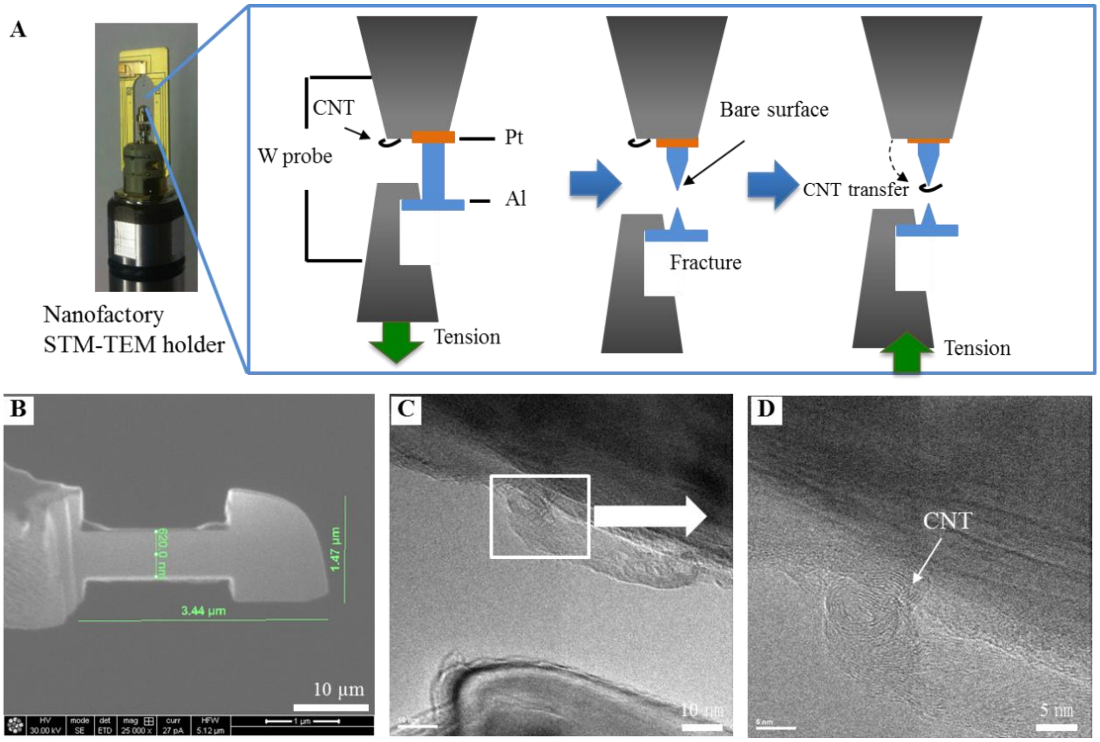

Fig. S8 Fabrication process of the oxide-free Al and transferring CNT onto the surface. (A) nanofactory holder and schematic diagram for preparing of the oxide-free Al and CNT transfer process. (B) SEM image after FIB. (C) TEM image after CNT transfer and (D) high-resolution TEM of the CNT wall.

For the comparison purpose, we pulled out the Al sample in the air to oxidize the surface. The CNT was transferred to the oxide passivated Al surface (Fig. S9A).

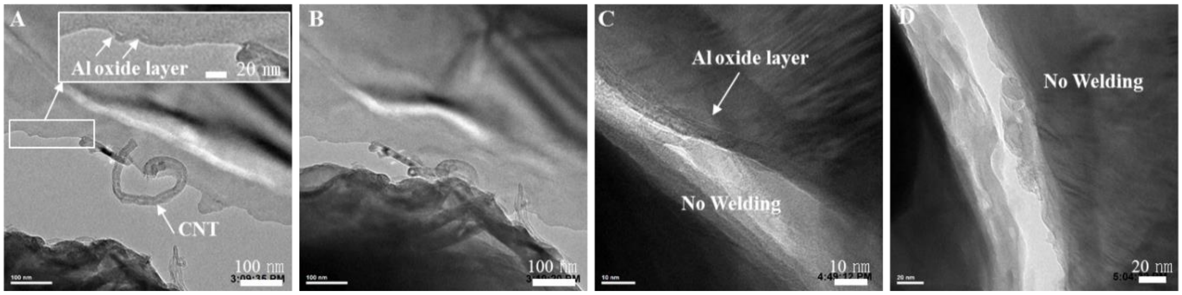

Fig. S9 Non-cold welding of the oxide layer-coated Al. (A) CNT on oxide coated-Al surface. (B) Contacting of two Als. The surface between two Als after (C) shearing and (D) impact.

When we contact the two Al, no atomically surface-diffusion binding between Al-Al was observed in the presence of the surface oxide as shown Fig. S9B and C. Therefore, the subsequent CNT embedding did not occur in the oxide formed Al (see Movie S4).

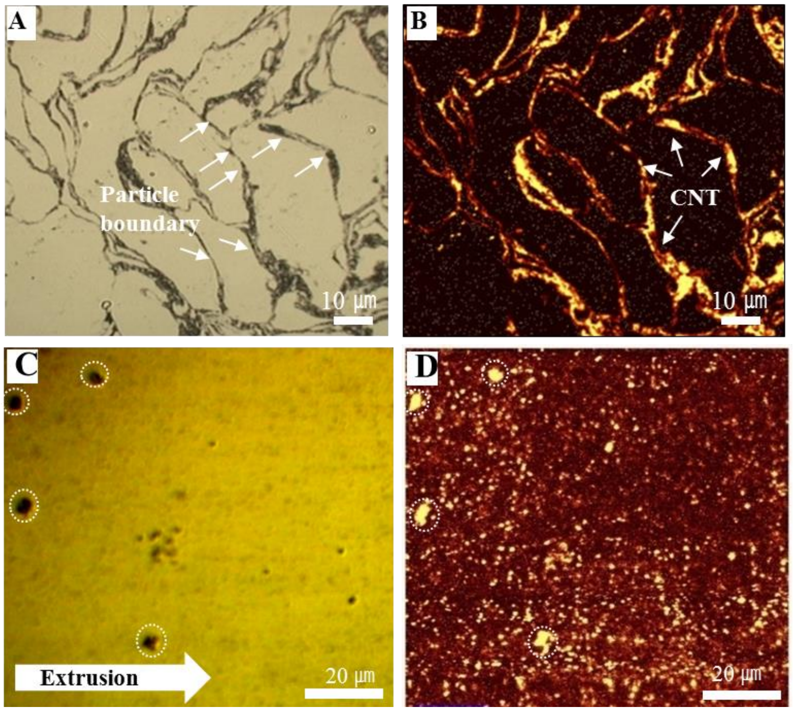

Fig. S10 Comparison of the dispersion of CNT after Al+CNT composite fabrication in oxidation/non-oxidation condition. (A) Optical image and (B) G band mapping in confocal Raman spectra at the oxidation condition. (C) Optical image and (D) G band mapping in confocal Raman spectra at the non-oxidation condition.

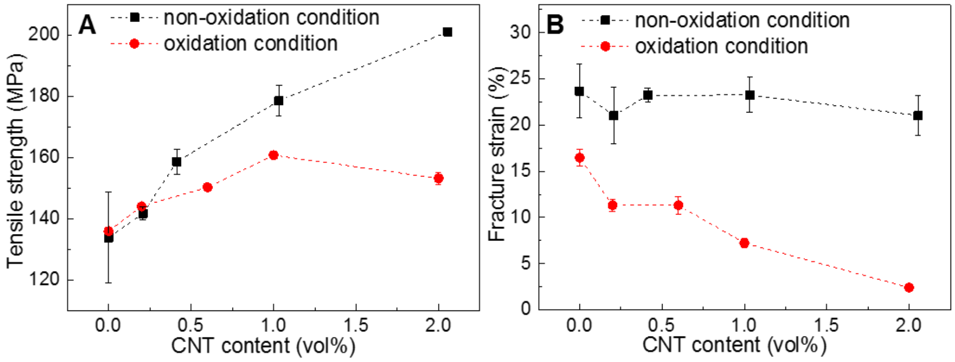

Fig. S11 The mechanical properties under different ambient conditions. A comparison of the mechanical properties with clustered CNTs: (A) tensile strength and (B) fracture strain as a function of the CNT concentration.

E. Atomistic diffusivity

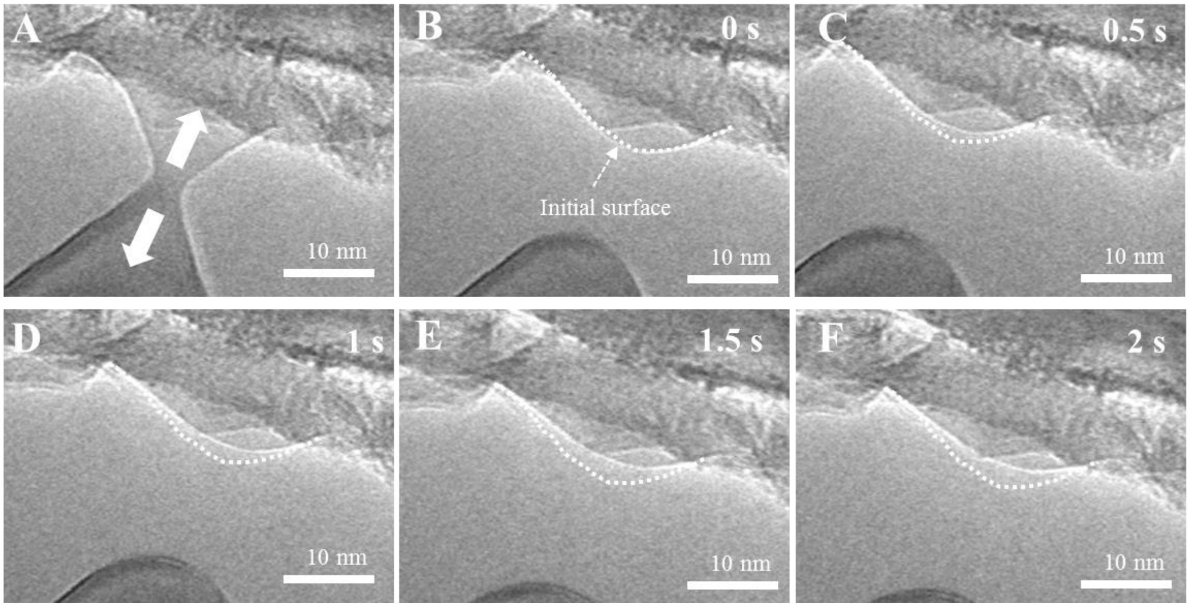

Fig. S12 flattening of the surface analysis after the massive atomic cluster transfer on the top of the CNT in in-situ TEM. (A) transferring of the Al, (B) 0s, (C) 0.5s, (D) 1s, (E) 1.5s and (F) 2s. white dot indicates the original surface of the Al.

The quantitative analysis of the surface diffusivity in in-situ TEM observation can be obtained by taking flattening time of a single-hump wrinkled surface after coverage of CNT via cold welding in Fig. S12. The surface diffusivity D_s can be estimated by the following equation.[5]

$$D_s = -\frac{S k_B T}{\nu \gamma_M \Omega^2}\left(\frac{\lambda}{2\pi}\right)^4$$

Where S is the slope from the natural logarithm of height vs a linear function of time in Fig. S4A. Ω is atomic volume (0.0166 nm^3/atom), T is room temperature (298 K), ν is the surface atomic density ($\nu_{(111)}$=0.1 atom/nm^2), and λ is the segment length in the rest position (24 nm in Fig. S13)

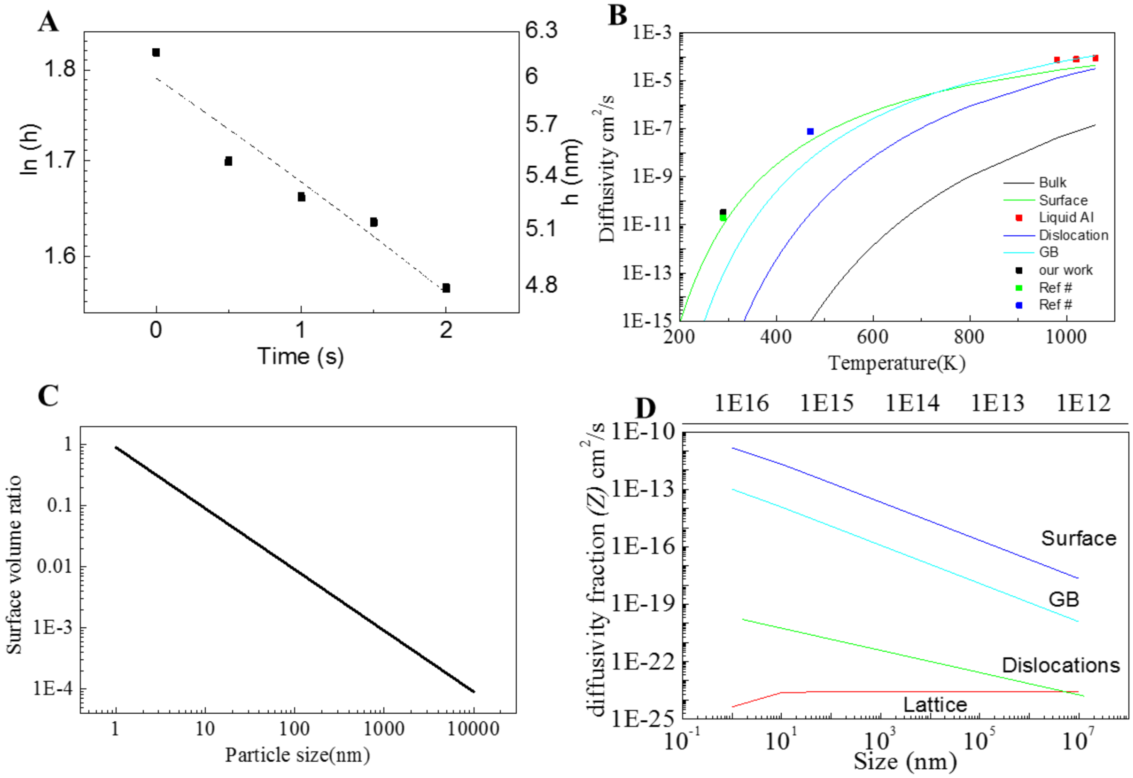

Fig. S13 (A) The measured retreating distance h on the surface CNT versus exposure time, (B) self-diffusivity of the atoms in Al versus temperature. (C) volume fraction of the surface to bulk volume (nm^3/nm^3). (D) Effective self-diffusivity of the Al atoms depending on the size according to the area fraction of surface, GB, dislocation. at room temperature.

Diffusivity of Al is extracted from the existing literature value. All the diffusion coefficient satisfy Arrhenius equation.

$$D = D_0 \exp(-\frac{Ea}{k_B T})$$

Where D_o is the pre-exponential factor, E_a is the activation energy, and k_B is the Boltzmann constant. The activation energy and pre-exponential factor were obtained from previous reported empirical value in Table S2.[6] The diffusivity of the surface, GB, dislocation, and bulk are plotted according to temperature change in Fig. S13B.

The ratio of surface volume fraction (V_s) to the bulk volume (V_b) was calculated by following equation.

$$\frac{V_s}{V_b} = \frac{t3\pi r^2}{\frac{4}{3}\pi r^3}$$

Where r is the diameter of particles and t is the thickness of top most surface (~0.3 nm). The ratio V_s/V_b is plotted in Fig. S13C

We have calculated the contribution of the diffusivity fraction (Z) in the entire system.

$$Z = Df$$

Where D is the original diffusivity of the surface, GB, dislocation, and bulk, f is the area fraction of its compound. t for GB was consider to be 0.3 nm. Z is plotted in Fig. S13B

Therefore, the effective diffusivity can be introduced from the sum of all the diffusivity component.

$$D_{Eff.} = Z_s + Z_{GB} + Z_{disl.} + Z_L$$

Table S3. Activation energy and diffusivity of Al[6]

Type	E_a (kcal/mol·K)	D_o (cm^2/s)	D at 298K(RT) (cm^2/s)	Fraction (f) at 10nm	Z (cm^2/s)
Surface	12.1	0.014	1.8E-11	0.1164	2.1E-12
GB	16.6	0.3	2.0E-13	0.0591	1.2E-14
Dislocation	23.3	2.1	1.6E-17	1E-3	1.6E-21
Bulk	32	0.5	2.7E-24	0.823	2.4E-24

B. *Measurements*

Microstructure characterization

The microstructure of the Al+CNT composites was characterized by FESEM and high-resolution TEM (HRTEM, 200 keV, 2100F, JEOL, Japan). The specimen for microstructure observation was prepared by a mechanical polishing (MetPrep3TM/PH-3TM, ALLIED, USA).

SiC paper (400~1200 grit) and alumina (3 μm and 1 μm) and silica (0.04 μm) suspensions were used to polish the specimen. The TEM sample was prepared using focused ion beam (FIB, SMI3050TB, SII, Japan) with a Ga ion milling process and a Pt protection layer. Elemental analysis was performed with energy-dispersive X-ray spectroscopy (EDS). The crystallographic analysis was performed using X-ray diffraction (XRD, Cu Kα, 1.54 Å, D/MAX 2500, Rigaku, Japan) and electron-backscattered diffraction (EBSD, EDAX). The EBSD patterns were selectively collected from orientation angles greater than 15°. The CNT distribution in the Al matrix was characterized using confocal Raman spectroscopy (CRM 200, Witech, German).

Mechanical properties measurement

The mechanical properties were characterized using an ultimate tensile tester (Landmark 25 kN, MTS, USA) and a micro-Vickers hardness tester (HM-211, Mitutoyo, Japan). The tensile specimen was prepared using a mechanical mill with a 6 mm gage diameter and a gage length of 25 mm (E8/E 8M-08, ASTM). The tension test was performed at a speed of 2 mm/min. The hardness test was performed on a cross-section of the specimen using a load of 100 g for 10 s.

Characterization of interfacial Al-C bonding

The interfacial Al-C bonds were characterized by XRD, Raman and XPS. The Al_4C_3 peaks in the XRD patterns were clearly visible with SPS processing at temperatures greater than 300 °C

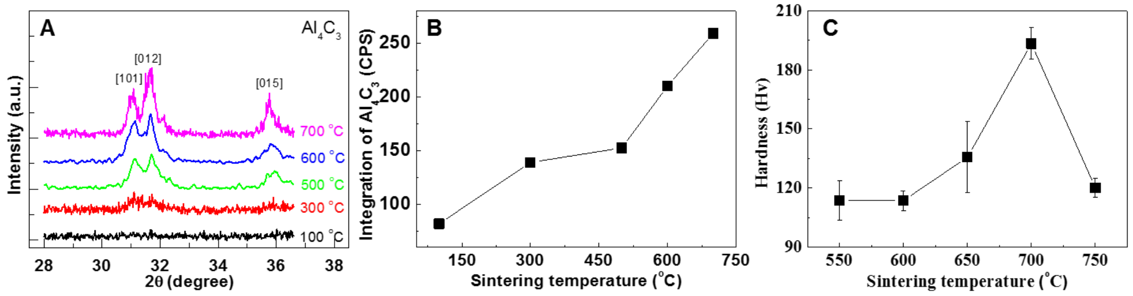

(Fig. S14A and B). The amount of the Al_4C_3 phase is proportional to the hardness of the composite (Fig. S14C). The increase of the hardness as a function of Al_4C_3 phase indicates the role of the interfacial Al-C covalent bonds for load transfer. The blueshift of the G-band peak position in the Raman spectra provides further evidence of the presence of Al-C covalent bonds in the composite[12], which is additionally supported by the C1s peak in the XPS spectrum (Fig. S15A and B). The frequently observed broken CNTs at fracture area are another evidence of the sufficient interfacial bonding.

Fig. S14 (A)XRD data for the samples in terms of the SPS temperature used to detect Al_4C_3 formation. (B) The formation of Al_4C_3 integrated by XRD and (C) the hardness of the Al+ 10 wt% CNT composite as a function of the sintering temperature. The formation of Al_4C_3 indicates strong interfacial strength between Al and CNT. Although the interfacial strength monotonically increased up to 700 °C with increasing sintering temperature, the hardness was degraded at high temperature (750 °C) due to CNT disintegration.

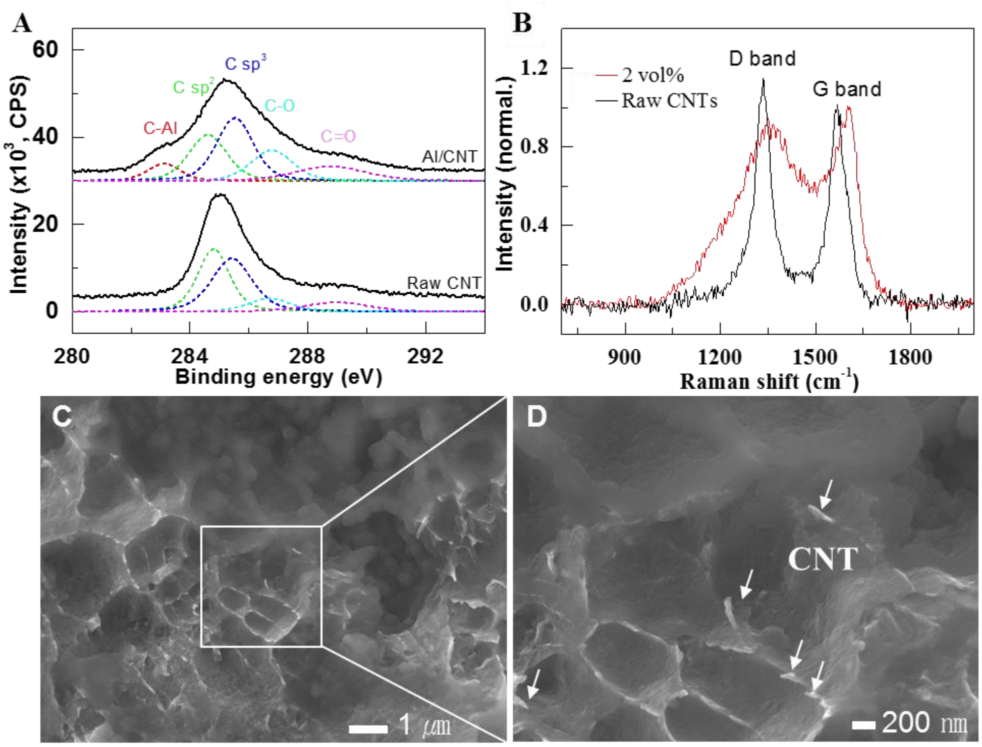

Fig. S15 Al-C interfacial covalent bonds. (A) XPS for C1s. The binding energy for carbon determined from the XPS spectrum of the Al/CNT 10 wt% composite after SPS process. A large volume percentage was used for the XPS measurements to clearly show the carbon peaks. (B) Raman spectra of raw CNT and 2vol% CNT in Al. The upshift of G band indicates charge transfer from CNT to Al through Al-C covalent bonds. SEM images of the fractured area after tension testing. (C) The Al/CNT 2 vol% composite and (D) protruding CNTs indicated by arrows, indicating efficient load transfer from the strong anchoring of Al to the CNT surface.

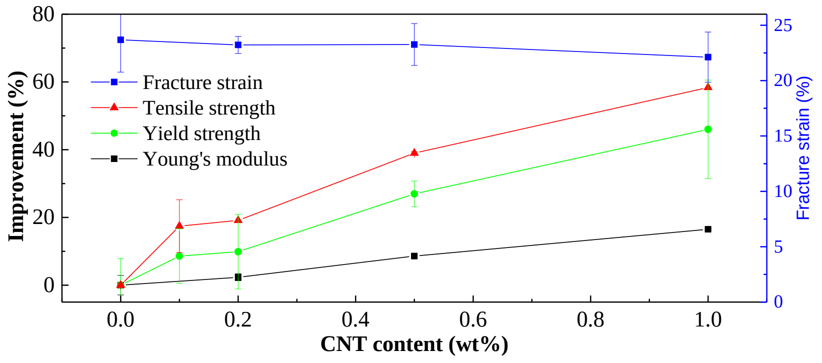

Fig. S16 Fracture strain and Relative improvement of Young's modulus, tensile strength and yield strength.

The specific strengthening efficiency (R)

The specific strengthening efficiency (R), $R = \frac{\sigma_c - \sigma_m}{\upsilon_c \sigma_m}$, where σ_c and σ_m are the tensile strengths of the composite and matrix, respectively, and υ_c is the volume percentage of CNTs to metal, is the relative tensile strength normalized by the CNTs vol% (Fig. S17A). The strengthening efficiency is generally low at high CNT content due to the limited dispersion ability. The specific strengthening efficiency (R) versus fracture strain curve clearly shows distinctive behavior at a CNT content of 2 vol%. The right region (with less than 2 vol% CNTs) shows samples with high fracture strain and high specific strengthening efficiency. In this region, the CNTs are well dispersed so that the number of Al-C bonds increases further and the dislocation propagation is effectively pinned, as shown in the right inset of Fig. S17B. However, in the left region (with more than 2 vol% CNT), both the fracture strain and the specific strengthening efficiency are substantially reduced. This trend is also well correlated with mechanical properties, such as the tensile strength and the Vickers hardness, for different CNT contents. Although SPS process produces interfacial Al-C bonds, the agglomerated CNTs provide less number of Al-C bonds due to the low interfacial area between Al and CNTs, resulting in inefficient dispersion hardening. The CNTs agglomeration at higher content eventually resulted in poor specific mechanical strength and fracture strain.

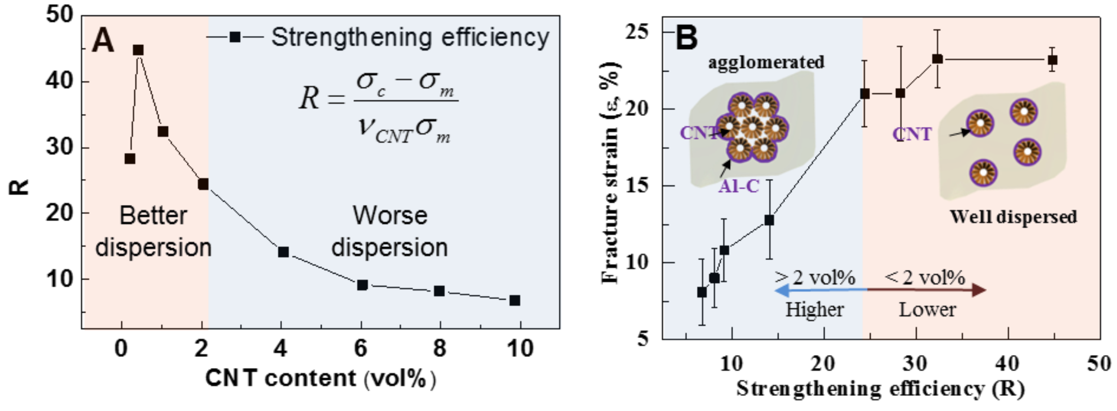

Fig. S17 (A) The fracture strain versus strengthening efficiency. Two distinct regions are clearly visible. The inset shows a schematic of degree of CNT dispersion.

Processing factors for affecting the specific toughness, specific strengthening efficiency, and specific fracture strain.

The toughness, ($\int_0^{\varepsilon_f} \sigma \, d\varepsilon$), is defined as a tolerance energy of absorbing external stress before a final fracture from the strain and stress curve. The toughness with respect to pure Al increases as the CNT content approaches to 2 vol% (Fig. 4). Our process provides greater toughness than previous wet processes.[7] This result implies that maintaining inert conditions by preventing Al oxidation to induce surface welding during mechanical pulverization is critical. At higher CNTs content (greater than 2 vol%(1wt%), the toughness gradually decreases until a value that is worse than that of pure Al. These results suggest that, in spite of enhanced mechanical properties by the incorporated CNTs, the agglomeration of CNTs significantly gives harmful effects for the ductility by provoking crack initiation.

Figure 5 summarizes the relationship of these properties with the material processing methods for the existing experimental data. As examples, the slurry mixing method caused the formation of a strong oxide layer on the surfaces of the Al particles, which resulted in poor CNTs dispersion, causing the low load transfer, the poor dislocation interactions with the CNTs, and the pore generations from the CNTs clusters.[8] Therefore, low strengthening efficiency, low toughness, and low fracture strain were obtained. In comparison, the low-energy ball milling (LEBM) process imparted mechanical impacts to the CNTs and provided the better CNTs dispersion than the solution processing.[9] Although this better dispersion results in an improved toughness as a consequence of maintaining the fracture strain, a low strengthening efficiency is observed due to the low load transfer from the poor interface. Strong interfacial bonding can be accommodated by *in-situ* CNTs growth on Al and improves load transfer, resulting in the greater strengthening efficiency without the loss of toughness and fracture strain.[10] High-energy ball milling (HEBM) generates highly dispersed CNTs and Al–C covalent bonds from the strong mechanical impact, improving the strengthening efficiency.[11,12] However, the structural damage of the CNTs cannot be avoided from the strong mechanical impact, and consequently, the poor

quality CNTs structure give a low specific toughness and fracture strain due to the inefficient pinning of dislocation propagation. The combination of ultrasonication and ball milling provides the better dispersion and a strong interface from the chemical functionalization without damage to the CNTs walls.[7,13-15] Therefore, the strengthening efficiency showed higher values than those achieved with LEBM. Nevertheless, the oxygen-containing solvent caused the oxidation of Al particles during the ultrasonication process, which rendered this method relatively inefficient with respect to the dispersion of CNTs into the Al grains. This again induced poor interaction between the CNTs and dislocations, which resulted in the low fracture strain. Our cold-welding induced mechanical dispersion of CNTs along with a strong interface yields a high degree of CNTs dispersion, even inside the Al grains, while minimizing the damage to the CNTs. Our method, therefore, provides not only high values of the strengthening efficiency and specific toughness but also a high specific fracture strain.

Movie S1 **http://li.mit.edu/S/KangPyoSo/Upload/microwavetreatment.wmv**

Movie S2 **http://li.mit.edu/S/KangPyoSo/Upload/meltingprocess.mpg**

Movie S3 **http://li.mit.edu/S/KangPyoSo/Upload/Movie1non-oxidewelding.wmv**

Movie S4 **http://li.mit.edu/S/KangPyoSo/Upload/MovieS1-oxidewelding.wmv**

References

1 So, K. P. *et al.* SiC formation on carbon nanotube surface for improving wettability with aluminum. *Compos Sci Technol* **74**, 6-13 (2013).

2 Y. H. Lee, K. P. S., H. K. Park, E. S. Kim. Method for increasing electro conductivity of aluminum using carbon nanotube and the material. Korea patent 10-1114628 (2012).

3 Xie, G. Q. *et al.* Effect of interface behavior between particles on properties of pure Al powder compacts by spark plasma sintering. *Mater Trans* **42**, 1846-1849, doi:DOI 10.2320/matertrans.42.1846 (2001).

4 Munir, Z. A., Anselmi-Tamburini, U. & Ohyanagi, M. The effect of electric field and pressure on the synthesis and consolidation of materials: A review of the spark plasma sintering method. *J Mater Sci* **41**, 763-777, doi:10.1007/s10853-006-6555-2 (2006).
5 Xie, D.-G. *et al.* In situ study of the initiation of hydrogen bubbles at the aluminium metal/oxide interface. *Nat Mater* **14**, 899 (2015).
6 Tan, C. M. & Roy, A. Electromigration in ULSI interconnects. *Materials Science and Engineering: R: Reports* **58**, 1-75 (2007).
7 Jiang, L., Li, Z. Q., Fan, G. L., Cao, L. L. & Zhang, D. The use of flake powder metallurgy to produce carbon nanotube (CNT)/aluminum composites with a homogenous CNT distribution. *Carbon* **50**, 1993-1998, doi:10.1016/j.carbon.2011.12.057 (2012).
8 Wu, J., Zhang, H., Zhang, Y. & Wang, X. Mechanical and thermal properties of carbon nanotube/aluminum composites consolidated by spark plasma sintering. *Materials & Design* **41**, 344-348 (2012).
9 Esawi, A. M. K., Morsi, K., Sayed, A., Gawad, A. A. & Borah, P. Fabrication and properties of dispersed carbon nanotube-aluminum composites. *Mat Sci Eng a-Struct* **508**, 167-173, doi:10.1016/j.msea.2009.01.002 (2009).
10 He, C. N., Zhao, N. Q., Shi, C. S. & Song, S. Z. Mechanical properties and microstructures of carbon nanotube-reinforced Al matrix composite fabricated by in situ chemical vapor deposition. *J Alloy Compd* **487**, 258-262, doi:10.1016/j.jallcom.2009.07.099 (2009).
11 Choi, H. J., Shin, J. H. & Bae, D. H. Grain size effect on the strengthening behavior of aluminum-based composites containing multi-walled carbon nanotubes. *Compos Sci Technol* **71**, 1699-1705, doi:10.1016/j.compscitech.2011.07.013 (2011).
12 So, K. P. *et al.* Low-temperature solid-state dissolution of carbon atoms into aluminum nanoparticles. *Scripta Mater* **66**, 21-24, doi:10.1016/j.scriptamat.2011.09.031 (2012).
13 Deng, C. F., Wang, D. Z., Zhang, X. X. & Li, A. B. Processing and properties of carbon nanotubes reinforced aluminum composites. *Mat Sci Eng a-Struct* **444**, 138-145, doi:10.1016/j.msea.2006.08.057 (2007).
14 Jiang, L., Li, Z. Q., Fan, G. L., Cao, L. L. & Zhang, D. Strong and ductile carbon nanotube/aluminum bulk nanolaminated composites with two-dimensional alignment of carbon nanotubes. *Scripta Mater* **66**, 331-334, doi:10.1016/j.scriptamat.2011.11.023 (2012).
15 Nam, D. H. *et al.* Synergistic strengthening by load transfer mechanism and grain refinement of CNT/Al-Cu composites. *Carbon* **50**, 2417-2423, doi:10.1016/j.carbon.2012.01.058 (2012).
